# Detection of Chronic Cognitive-Motor Deficits in Adults With a History of Concussion Using Computerized Eye-Hand Coordination Tasks: Preliminary Experimental Design Study

**DOI:** 10.2196/70867

**Published:** 2025-08-05

**Authors:** Qin Zhu, Shaochen Huang

**Affiliations:** 1 Division of Kinesiology and Health, University of Wyoming, Laramie, WY, United States; 2 School of Health Promotion and Kinesiology, Texas Woman's University, 1600 N Bell Ave, Pioneer Hall, Denton, TX, 76209, United States, 1 940-898-2592

**Keywords:** concussion screening, coordination, cognitive-motor functions, perception-action coupling, eye-hand coordination

## Abstract

**Background:**

Concussion has been a major public health concern due to the substantial cognitive sequelae it results. Although the dysfunctions of the frontal lobe and corpus callosum owing to concussions have been documented, the existing concussion screening tools merely examine cognitive functions in isolation of motor functions and failed to detect the chronic cognitive-motor impairments following concussions. Yet, there has been no concussion screening test aimed specifically to assess the coupled cognitive-motor functions.

**Objective:**

This study aimed to provide preliminary evidence for using computerized eye-hand coordination tasks to detect chronic cognitive-motor deficits associated with concussion history.

**Methods:**

The computerized eye-hand coordination tasks were used to assess the coupled cognitive-motor functions of the participants with and with no history of concussion. In experiment 1, a total of 12 participants (6 young adults with a history of concussion and 6 healthy controls) completed longitudinal assessments of coordination profiles across a year. Experiment 2 examined a total of 20 participants (10 participants with a history of concussion and 10 healthy controls) using an iterated single-session protocol. *Just noticeable difference* (JND) and *proportion of time-on-task* (PTT) were used to assess cognitive-motor performance. Mixed-design ANOVAs were used to examine group differences, and the effect sizes were assessed using Cohen *d* test.

**Results:**

In experiment 1, participants with a history of concussion exhibited more inconsistent ability to visually discriminate the in-phase coordination pattern (coefficient of variation of JND: participants with a history of concussion = mean 0.27, SD 0.04, and healthy controls = mean 0.17, SD 0.07; *t*_10_=2.93; *P*=.02). Similarly, their performance on unimanual and bimanual in-phase and anti-phase coordination patterns was significantly poorer (at in-phase: PTT_Concussed_=mean 0.63, SD 0.10, and PTT_Healthy_=mean 0.73, SD 0.08 [*F*_1,10_=8.49; *P*=.02]; at anti-phase: PTT_Concussed_=mean 0.46, SD 0.14, and PTT_Healthy_=mean 0.60, SD 0.10 [*F*_1,10_=10.67; *P*=.008]). In experiment, 2 where only the unimanual coordination tasks were implemented for screening, participants with a history of concussion showed impaired performance in both in-phase and anti-phase tasks (at in-phase: PTT_Concussed_=mean 0.62, SD 0.13, and PTT_Healthy_=mean 0.74, SD 0.07 [*F*_1,54_=4.20; *P*=.045]; at anti-phase: PTT_Concussed_=mean 0.37, SD 0.15, and PTT_Healthy_=mean 0.56, SD 0.14 [*F*_1,54_=10.26; *P*=.002]), and they also failed to show the differentiated performance between anti-phase and 90° coordination patterns (PTT_Anti-phase_=mean 0.37, SD 0.15, and PTT_90° coordination_=mean 0.37, SD 0.13).

**Conclusions:**

Due to their ability to detect both impaired and undifferentiated performance in producing intrinsic and novel coordination patterns, the unimanual coordination tasks appear to be a sensitive screening tool for chronic cognitive-motor deficits associated with history of concussion.

## Introduction

Most concussions fall in the category of mild traumatic brain injury (mTBI) [1,2]. More than 81% of the 69 million traumatic brain injuries that occur annually worldwide are considered mTBI [3]. Despite the label of “mild,” individuals with concussion often experience cognitive, physical, psychological, and social dysfunctions that severely impact the quality of their lives, causing huge social and economic burdens [4-6]. Although individuals who experienced concussions could recover spontaneously within a few weeks to months, persisting symptoms and permanent deficits could remain as severe sequelae [7]. Thus, the National Institute of Neurological Disorders and Stroke and the National Institutes of Health declared concussion as a major public health concern, and efforts to improve early diagnosis and reduce postconcussion disability should be one of the national research priorities [8,9].

The frontal lobe is known as a brain area where the primary motor cortex (M1) is located and responsible for both cognitive and motor functions (eg, attention, memory, action planning, and decision-making). It has been reported that the functions of the frontal lobe are significantly impaired following a severe or moderate traumatic brain injury [10,11] or with enduring multiple mTBI [12,13]. Although brain atrophy could result from multiple concussions [14], clinical neuroimaging is not routinely performed for a reported case of concussion due to the high cost and the likelihood of undetected brain structural damage [15,16]. Therefore, numerous neurobehavioral assessment tools have been developed to examine the frontal lobe dysfunctions associated with concussion. For instance, the Sport Concussion Assessment Tool is a standardized screening tool that has been widely adopted for immediate assessment of a sport-related concussion, which consists of a series of tasks to assess cognitive (eg, attention and memory) and motor (eg, movement speed or accuracy and balance) functions of athletes. Although effective, the existing screening tools of concussion have two limitations. First, they are insensitive to capture the subacute and chronic cognitive-motor impairments following concussions. A scoping review [17] showed that a large proportion of individuals with a single concussion continued to demonstrate the measurable cognitive impairment, whereas the standard neurobehavioral assessments indicated no cognitive impairments. Accordingly, Sport Concussion Assessment Tool 5th edition has been recommended to be inappropriate for assessing people in the subacute and chronic phases of injury [18]. Second, they examine the cognitive functions in isolation with the motor functions, which consequently increases testing time and decreases efficiency due to the need of integrating different testing scores for overall rating. Given the high integration of cognitive and motor functions in the frontal lobe [19], assessing the coupled cognitive-motor functions should be more straightforward and efficient than the uncoupled approaches, which unfortunately have been missing in the current neurobehavioral screen tools for concussion.

Coordination is required for many functional motor tasks such as walking and running. The brain areas involved in performing and learning coordination tasks are the frontal lobe and cerebellum [20,21]. Research suggests an important link between coordination capability and cognitive-motor functions. Young adults diagnosed with developmental coordination disorder often demonstrate deficits in performing tasks that demand attention, working memory, or cognitive flexibility [22]. Due to its high relevance to cognitive-motor functions, coordination tasks including finger-to-nose test [23], the visuomotor tracking task [24], the divided-attention gait task [25], and bimanual coordination task [26] are often included in neurobehavioral screen tools for concussion. Meanwhile, many studies [27,28] have reported that the brain’s largest white matter structure, corpus callosum (CC), is vulnerable to concussion. Since CC is known for its function in interhemispheric processing, the structural damage of CC from concussions is associated with the impaired coordination performance demonstrated by the participants with a history of concussion, which makes coordination task alone as a decent screening tool for concussion. However, in using coordination tasks to assess cognitive-motor dysfunctions associated with concussion, the coordination task is often treated as a motor task with a variety of performance outcome measures (eg, speed, accuracy, motion asymmetry, motion continuity, or pattern stability). In view of the perception-action theory of coordination [29,30], the ability to coordinate limb movements is coupled with the ability to perceive the coordination pattern. Therefore, a comprehensive coordination assessment should involve both performing and perceiving the coordination pattern. Yet, there has been no concussion screening test aimed to assess the coupled cognitive-motor functions associated with coordination.

In this study, we explored whether the computerized eye-hand coordination tasks, implemented to assess the coupled cognitive-motor functions, could be used to detect the chronic and subacute cognitive-motor deficits associated with history of concussion. Three different coordination tasks were implemented through human-computer interaction to assess people’s ability to both perform and perceive different coordination patterns. Coordination performance was assessed by the established measures in psychophysical studies [31-33]. Adult participants with a history of concussion were recruited and gender-matched with healthy controls in 2 experiments, and their coordination performance was compared to determine whether a specific coordination task has the power to detect the group difference. Three coordination tasks (visual discrimination, unimanual, and bimanual) were implemented in experiment 1 to detect the group difference longitudinally and determine the sensibility of task and task measures for screening. We hypothesized that the impaired cognitive-motor functions could be detected by the respective task measures.

Experiment 2 was then followed to determine whether a simplified coordination screening test was sufficient to differentiate people with a history of concussion from those with no history of concussion in a single testing session. Based on the findings from experiment 1, we adjusted the testing protocol to assess participants’ performance of 3 coordination patterns (in-phase, 90^○^, and anti-phase) in just 1 testing session. Each pattern was examined by a bout of five 20-second-long trials. Such an adjustment was made to examine whether the unimanual coordination task could serve as a quick and efficient screening tool for concussion. It was hypothesized that the performance of 3 coordination patterns would differ between participants with a history of concussion and healthy participants.

## Methods

### Experiment 1

#### Participants

Six young adults with a history of concussion (mean age 22.00, SD 4.34 years; mean time from last concussion 38.67, SD 31.94 months: 2 males and 4 females) and 6 gender-matched healthy controls with no history of concussion (mean age 20.83, SD 2.64 years) were recruited on campus at the University of Wyoming. All concussions were medically diagnosed with accessible records. All participants had no history of substance abuse (drugs, alcohol, or controlled medication), peripheral neuropathy (including loss or decreased sensation or motor function in the upper extremity), or any neurological disorders that could impair coordination abilities (such as ataxia, giant axonal neuropathy, or multiple sclerosis).

#### Ethical Considerations

This study was approved by the institutional review board at the University of Wyoming (protocol number 20170220SH01461). All participants provided written informed consent prior to participation and were informed of their right to withdraw from the study at any time without penalty. All data collected were deidentified prior to analysis to ensure participant privacy and confidentiality. No personally identifiable information was stored. No monetary compensation was provided to participants for their participation in this study.

#### Apparatus

A joystick-computer interaction system was used for implementation of the visual discrimination, unimanual, and bimanual coordination tasks. Participants sat on a stool that could be adjusted for height to see a laptop screen at their eye-height level. A 15" PC laptop with a screen resolution of 1024×768 and a refresh rate of 60 Hz was set on top of a custom-built shelf facing the participant. Two Logitech Force 3D joysticks were connected via USB to the PC laptop, one opposite the left shoulder of the participant and the other opposite the right shoulder. Participants manipulated both joysticks in performing the bimanual task, while only the right joystick was used in performing the unimanual tasks. None of the joysticks was used for the visual discrimination task. The joysticks were hidden underneath by a tablecloth covering the shelf. All participants were able to reach and grasp the joysticks comfortably without seeing them.

**Figure 1.**
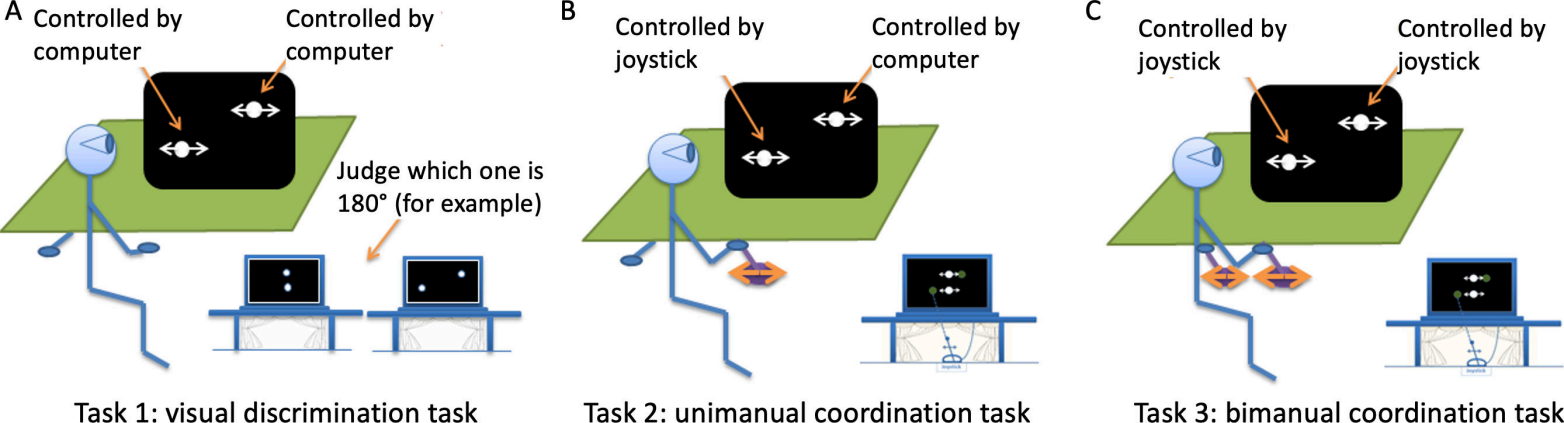
An illustration of experimental setting for (A) visual discrimination task; (B) unimanual coordination tasks, and (C) bimanual coordination tasks. Visual feedback about the correctness of the produced pattern was provided by changing the color of dots to green in unimanual and bimanual tasks.

The computer displayed 2 white dots against a black background on screen, one above the other. In the visual discrimination task, both dots were controlled by the computer, and participants were asked to watch the computer displays and make the judgments by pressing the assigned keys. In the unimanual task, participants controlled only the bottom dot via the right joystick, while the top dot was controlled by the computer. Finally, in the bimanual coordination task, the top dot was controlled by the left joystick and the bottom by the right joystick all the time (see Figures 1A-1C for experimental setting and tasks). The movement amplitude of the dots was 300 pixels, and they were each 60 pixels in diameter. The viewing distance was 70 cm, yielding an oscillating movement spanned at approximately 7.5° visual angle, equivalent to the movement amplitude of 18 cm in the horizontal plane on the joysticks. Stimulus presentation, data recording, and all data analyses were handled by a custom MATLAB toolbox written by ADW incorporating the Psychtoolbox [34].

#### Procedure

All participants were assessed by the following 3 eye-hand coordination tasks in a year period with 6 assessments separated by 2 months. The experimental procedures were designed to assess participants’ visual perception and motor coordination abilities.

##### Visual Discrimination Task

Participants performed a 2-alternative forced choice judgment task with a computer. Each trial started with a 4-second visual demonstration of a target rhythmic pattern (2 dots kept oscillating to one another) on the screen, followed by a pair of 4-second visual displays of rhythmic patterns for judgment: one being the target pattern and the other the distracting pattern. Both target and judgment patterns were played at 0.75 Hz. Participants must tell which 1 of the 2 presented judgment patterns was the target pattern by pressing a key: “A” for the first presented pattern, and “L” for the second presented pattern. Two intrinsic coordination patterns (in-phase and anti-phase) were used as target patterns. The in-phase pattern showed 2 dots moving in the same direction at the same speed, while the anti-phase pattern showed 2 dots moving in the opposite direction at the same speed, both in the top-bottom configuration. The number of trials was determined using 2 independent but interleaved transformed staircase procedures (see details about the staircase procedure in section “Data Analysis”). Thus, the total number of trials and duration of the task varied for each participant.

##### Unimanual Coordination Task

Participants performed 36 trials of unimanual joystick task with the joystick-computer interaction system, including 3 trials at the in-phase pattern, 3 trials at the anti-phase pattern, and 30 trials at a novel coordination pattern (90° relative phase, ie, half of the time the 2 dots move in the same direction and half of the time they move in the opposite direction). For each pattern, participants started with a 5-second visual demonstration of a target coordination pattern at 0.75 Hz, followed by trials (10 seconds each) in which they controlled 1 joystick to move 1 dot on the screen together with another dot controlled by the computer to replicate the target coordination pattern. Visual feedback about the correctness of the produced 90° coordination pattern was provided by changing the color of dots to green.

##### Bimanual Coordination Task

Similar procedures were followed as in the unimanual coordination task. However, instead of controlling 1 joystick, participants controlled 2 joysticks to move the 2 dots on the screen in order to replicate the target coordination pattern. Two intrinsic coordination patterns (ie, in-phase and anti-phase) and the novel coordination pattern were assessed with visual feedback provided only for the latter.

In this study, we were specifically interested in participants’ ability to learn a novel coordination pattern with visual feedback. As numerous studies have shown that in-phase and anti-phase are intrinsic coordination patterns that healthy people can produce readily [35,36], while 90° coordination is challenging and requires a significant amount of training to produce [37], we provided more trials and visual feedback about the correctness for participants to perform 90° coordination in both unimanual and bimanual tasks.

### Data Analysis

The staircase method was used to calculate the *just noticeable difference* (JND) in the visual discrimination task. The staircase is a psychophysical procedure for estimating perceptual thresholds, which makes the judgments harder or easier as a function of the correctness of the last trials. In this study, the magnitude of distracting patterns (the difference between the 2 displayed coordination patterns) was determined by a 1-up or 2-down rule defined in the staircase method. Considering the time constraint for a screening task and based on a previous study on the perception of coordination patterns [38], we applied a step size “up” of 1° and a stop rule of 3 reversals (errors) to the in-phase, and a step size “up” of 5° and a stop rule of 2 reversals (errors) to the anti-phase. Step size “down” was fixed based on Table 5.1 of Kingdom and Prins [39]. Thus, it was 0.55° for in-phase and 2.74° for anti-phase. The initial difference was set to 5° and 40° for in-phase and anti-phase, respectively. Each time the trial stepped down until the first error occurred, then it repeated until meeting the stop rule. In addition, the *coefficient of variation* (CV) was calculated for JNDs collected from the 6 assessments, respectively, for in-phase and anti-phase patterns to examine how consistent the participant was able to discriminate intrinsic coordination patterns.

In unimanual and bimanual coordination tasks, the *proportion of time-on-task* (PTT) was automatically calculated by the system and used to quantify the coordination performance. Specifically, the proportion of each continuous relative phase time series (trial) that fell within the range of the target phase ±20° tolerance was computed. PTT has been proven as a valid measure of performance at the required relative phase that can capture both accuracy and stability of coordination performance in numerous studies [32,33,40].

To examine the group difference in each of the eye-hand coordination tasks across the 6 testing sessions, the mixed-design ANOVA was performed on JNDs in the visual discrimination task and mean PTTs in both unimanual and bimanual coordination tasks, respectively, treating group (group with a history of concussion vs healthy controls) as a between-subject variable and testing sessions (1 through 6) as a within-subject variable. Prior to conducting ANOVAs, the normality of outcome variables was assessed using the Shapiro-Wilk test and all variables met the normality assumption. For post hoc pairwise comparisons following the detection of significant ANOVA effect, we applied Tukey HSD (Honestly Significant Difference) corrections to adjust for multiple comparisons. Since the study purpose was to examine the use of eye-hand coordination tasks to detect the postconcussion cognitive-motor deficits, we were more interested in the main effect of group, and the main effect of testing sessions would indicate only the learning effect associated with participants being familiar with the assessment tasks. In addition, the 2-tailed independent samples *t* test was performed on CV of JND at in-phase and anti-phase patterns, respectively.

Finally, due to the small sample size that might have limited the power of the study to detect the effects of interest, Cohen *d* effect size was calculated for each investigated factor. According to Cohen [41], *d*=0.20, *d*=0.50, and *d*=0.80 were considered as “small,” “medium,” and “large” effect sizes, respectively. A large effect size would indicate a strong effect to be detected in the future by a larger sample size.

### Experiment 2

#### Participants

Another 10 young adults with a history of concussion (mean age 20.90, SD 1.45 years; mean time from last concussion 23.88, SD 17.04 months: 6 males and 4 females) and 10 gender-matched healthy controls (mean age 22.30, SD 1.70 years) were recruited on campus at the University of Wyoming. The inclusion and exclusion criteria were identical to those used in the first experiment.

#### Ethical Considerations

This study was approved by the institutional review board at the University of Wyoming (protocol number 20180425SH01973). The same informed consent process, privacy protections, and compensation policies applied as in experiment 1.

#### Apparatus and Procedure

Only the unimanual coordination task was used, and participants were tested with 3 coordination patterns (in-phase, anti-phase, and 90° coordination) using the same apparatus as in experiment 1. Participants performed 1 bout of 5 trials for each pattern. Each bout was started with a 10-second visual demonstration of a target coordination pattern at 0.75 Hz, followed by five 20-second trials of replicating the target pattern by moving 1 joystick to move 1 dot on the screen together with another dot controlled by the computer. No visual feedback was provided during each trial.

#### Data Analysis

PTT was calculated to quantify the eye-hand coordination performance at each coordination pattern. A mixed-design ANOVA was performed on mean PTT data, treating group (participants with a history of concussion vs healthy controls) as a between-subject variable, and target coordination pattern (in-phase vs 90° vs anti-phase) as a within-subject variable. Prior to conducting ANOVAs, the normality of outcome variables was assessed using the Shapiro-Wilk test, which showed that all variables met the normality assumption. Cohen *d* effect size was again calculated to demonstrate the power of the study due to the small sample size.

## Results

### Experiment 1

#### Visual Discrimination of Coordination

No group difference was detected for JND at either in-phase or anti-phase (in-phase: *F*_1,10_=1.11, *P*=.32; anti-phase, *F*_1,10_=0.48, *P*=.50), indicating that the threshold for identifying the in-phase and anti-phase coordination pattern was similar between the participants with a history of concussion (JND_in-phase_=mean 1.53°, SD 0.46°; JND_anti-phase_=mean 14.29°, SD 4.87°) and the healthy participants (JND_in-phase_=mean 1.39°, SD 0.29°; JND_anti-phase_=mean 13.19°, SD 4.26°). The effect of testing sessions was detected only for JND of anti-phase pattern (*F*_5,50_=3.01; *P*=.02), suggesting that both groups increased their ability to discriminate anti-phase coordination patterns in later testing sessions (see Table 1 for test means).

**Table 1.** Test means for all task measures across 6 tests (mean, SD) in experiment 1*.*

	Test 1	Test 2	Test 3	Test 4	Test 5	Test 6
JND^a^ (degree)						
JND of in-phase	1.66° (0.47°)	1.54° (0.34°)	1.32° (0.36°)	1.30° (0.30°)	1.45° (0.38°)	1.49° (0.42°)
JND of anti-phase	16.80° (4.59°)^b^	14.96° (5.06°)	12.64° (3.57°)	13.80° (5.53°)	11.53° (3.99°)	12.71° (3.09°)
Mean proportion of time-on-task						
Unimanual in-phase	0.61 (0.08)^c^	0.65 (0.13)	0.68 (0.09)	0.71 (0.07)	0.71 (0.09)	0.74 (0.08)
Unimanual anti-phase	0.46 (0.14)	0.50 (0.13)	0.53 (0.19)	0.59 (0.11)	0.56 (0.14)	0.57 (0.11)
Unimanual 90°	0.33 (0.09)^d^	0.43 (0.13)	0.47 (0.13)	0.49 (0.13)	0.52 (0.13)	0.55 (0.14)
Bimanual in-phase	0.70 (0.05)	0.71 (0.06)	0.75 (0.04)	0.74 (0.04)	0.76 (0.04)	0.72 (0.06)
Bimanual anti-phase	0.82 (0.04)	0.84 (0.04)	0.82 (0.04)	0.82 (0.04)	0.81 (0.07)	0.83 (0.04)
Bimanual 90°	0.30 (0.08)^e^	0.37 (0.09)^f^	0.45 (0.06)	0.48 (0.09)	0.45 (0.11)	0.49 (0.12)

a JND: just noticeable difference.

b Test 1 vs test 5, *P*=.05.

c Test 1 vs test 6, *P*<.05.

d Test 1 vs test 4, *P*<.05; test 1 vs test 5, *P*<.01; and test 1 vs test 6, *P*<.01.

e Test 1 vs test 3, *P*<.01; test 1 vs test 4, *P*<.01; test 1 vs test 5, *P*<.01; and test 1 vs test 6, *P*<.01.

f Test 2 vs test 6, *P*<.05.

**Figure 2.**
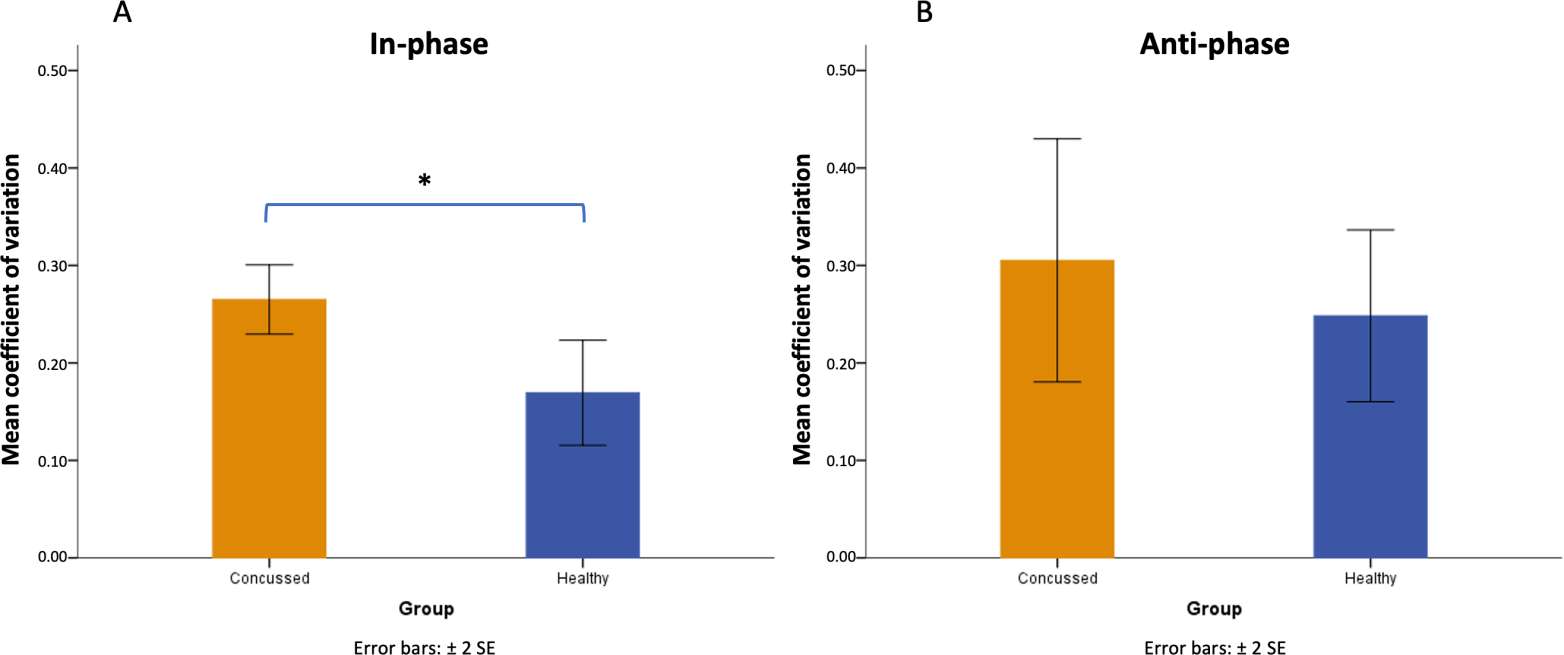
Coefficient of variation of just noticeable difference at in-phase (panel A) and anti-phase pattern (panel B) for participants with a history of concussion and healthy controls. *Significant difference with *P*<.05*.* Error bars represent the standard error of the mean.

The group difference was detected on CV of JND for in-phase coordination pattern (*t*_10_=2.93; *P*=.02) but not for anti-phase coordination pattern (*t*_10_=0.77; *P*=.46). As seen in Figures 2A and 2B, participants in the group with a history of concussion demonstrated a higher inconsistency in their ability to discriminate the in-phase coordination pattern than the healthy controls across the 6 assessments (CV_Concussed_=mean 0.27, SD 0.04; CV_Healthy_=mean 0.17, SD 0.07), which was not seen in discriminating the anti-phase coordination pattern. No other main effect or interaction was detected (all other *P* values >.05).

#### Unimanual Coordination Performance

**Figure 3.**
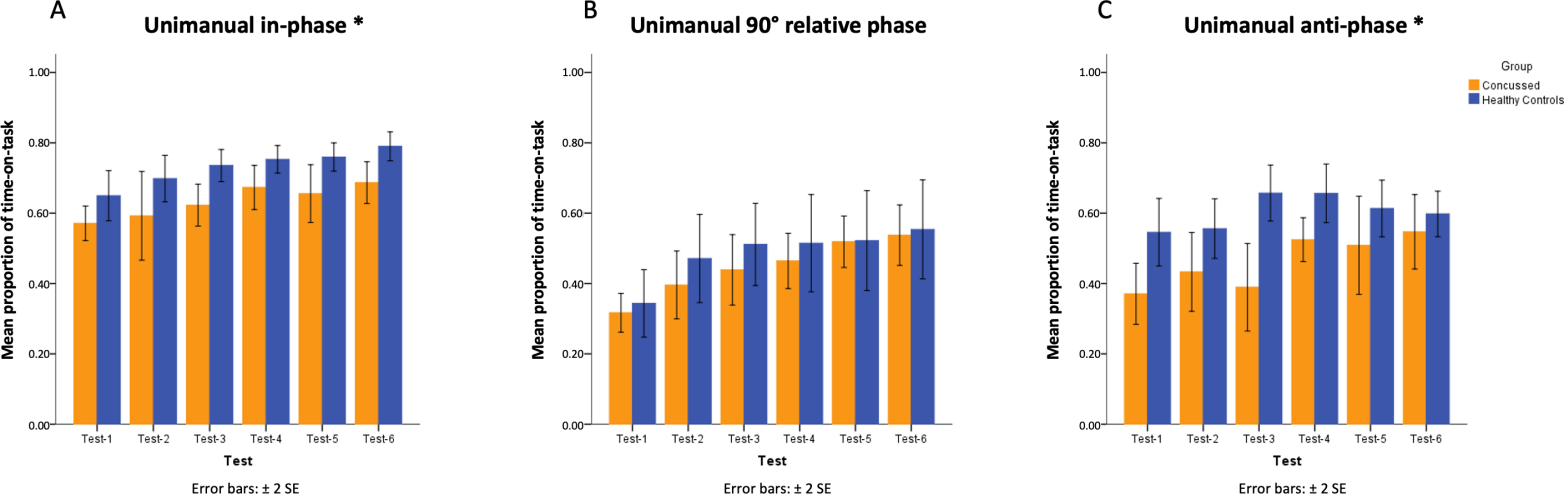
Mean proportion of time-on-task at unimanual in-phase (panel A), 90° relative phase (panel B), and anti-phase pattern (panel C) for participants with a history of concussion and healthy controls across 6 tests. *Group main effect revealed by mixed-design ANOVA. Error bars represent the standard error of the mean.

The main effect of group was detected for performing both in-phase and anti-phase coordination patterns (at in-phase: *F*_1,10_=8.49, *P*=.02; at anti-phase: *F*_1,10_=10.67, *P*=.008; Figure 3A-C). Healthy controls overall outperformed participants with a history of concussion in producing the intrinsic coordination patterns (at in-phase: PTT_Concussed_=mean 0.63, SD 0.10, and PTT_Healthy_=mean 0.73, SD 0.08; at anti-phase: PTT_Concussed_=mean 0.46, SD 0.14, and PTT_Healthy_=mean 0.60, SD 0.10). The main effect of testing sessions was detected in performing both intrinsic coordination patterns as well (at in-phase: *F*_5,50_=7.03, *P*<.001; at anti-phase: *F*_5,50_=3.16, *P*=.014). In general, healthy controls and participants with a history of concussion both improved their performance of intrinsic coordination patterns in later testing sessions, suggesting that the unimanual coordination task is challenging even for producing the intrinsic coordination patterns (see Table 1 for test means).

The main effect of testing sessions was detected in performing the novel coordination pattern at the 90° relative phase (*F*_5,50_=18.92; *P*<.001). Regardless of the history of concussion, all participants learned and improved their unimanual 90° coordination. No other main effect or interaction was detected (all other *P* values >.05).

#### Bimanual Coordination Performance

**Figure 4.**
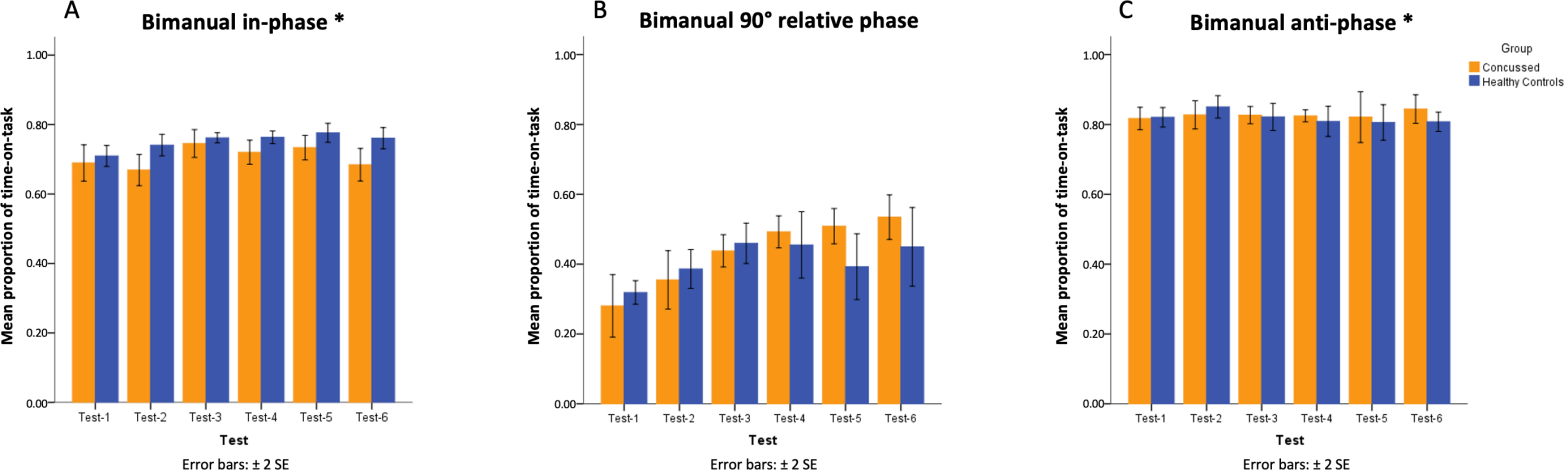
Mean proportion of time-on-task at bimanual in-phase (panel A), 90° relative phase (panel B)*,* and anti-phase pattern (panel C) for participants with a history of concussion and healthy controls across 6 tests*.* *Group main effect revealed by mixed-design ANOVA. Error bars represent the SE of the mean.

The main effect was detected for both group (*F*_1,10_=6.49; *P*=.03) and testing sessions (*F*_5,50_=6.15; *P*<.001) in performing the in-phase coordination pattern with no interaction (*F*_5,50_=1.65; *P*=.16). As seen in Figures 4A-4C, participants with a history of concussion performed the in-phase coordination pattern worse than healthy controls (PTT_Concussed_=mean 0.71, SD 0.06; PTT_Healthy_=mean 0.75, SD 0.04), and both groups improved their performance throughout the testing sessions (Table 1). No main effect or interaction was detected in performing the anti-phase coordination pattern (all other *P* values >.05).

As for the performance of 90° coordination, the significant main effect of testing session (*F*_5,50_=19.02; *P*<.001) and the group by testing session interaction (*F*_5,50_=3.81; *P*=.005) were detected, but the group difference was insignificant (*F*_1,10_=0.35; *P*=.57). Although participants improved their performance at 90° across the testing sessions, their performance changed differently depending on the group. While the health controls dropped their performance in later sessions, participants with a history of concussion maintained their improved performance in later sessions. As shown by the following simple main effect analysis with post hoc of Tukey HSD analysis, participants with a history of concussion outperformed the healthy controls only in the fifth session (PTT_Concussed_=mean 0.51, SD 0.06; PTT_Healthy_=mean 0.39, SD 0.12).

#### Cohen *d* Effect Size of All Coordination Tasks

As seen in Table 2, a large effect size (*d*>0.80) was detected for CV of JND in discriminating the in-phase coordination pattern, PTT in performing the unimanual in-phase and anti-phase coordination tasks, and PTT in performing the bimanual in-phase coordination task, suggesting that these tasks and task measures are promising for screening participants with a history of concussion.

**Table 2.** Cohen *d* effect size for all task measures in experiment 1*.*

Task measures	Main effect		Interaction	Cohen *d* of group main effect
JND^a^ of in-phase	N/A^b^	N/A	N/A	0.689
JND of anti-phase	N/A	Test	N/A	0.373
CV^c^ of JND at in-phase	Group	N/A	N/A	*1.362* ^d^
CV of JND at anti-phase	N/A	N/A	N/A	0.493
PTT^e^ of unimanual in-phase	Group	Test	N/A	*1*.591
PTT of unimanual anti-phase	Group	Test	N/A	*1.787*
PTT of unimanual 90°	N/A	Test	N/A	0.314
PTT of bimanual in-phase	Group	Test	N/A	*1.489*
PTT of bimanual anti-phase	N/A	N/A	N/A	0.251
PTT of bimanual 90°	N/A	Test	Group × Test	0.316

a JND: just noticeable difference.

b N/A: not applicable.

c CV: coefficient of variation.

d Tasks with over large effect size (Cohen *d*>0.80) are in italics.

e PTT: proportion of time-on-task.

### Experiment 2

**Figure 5.**
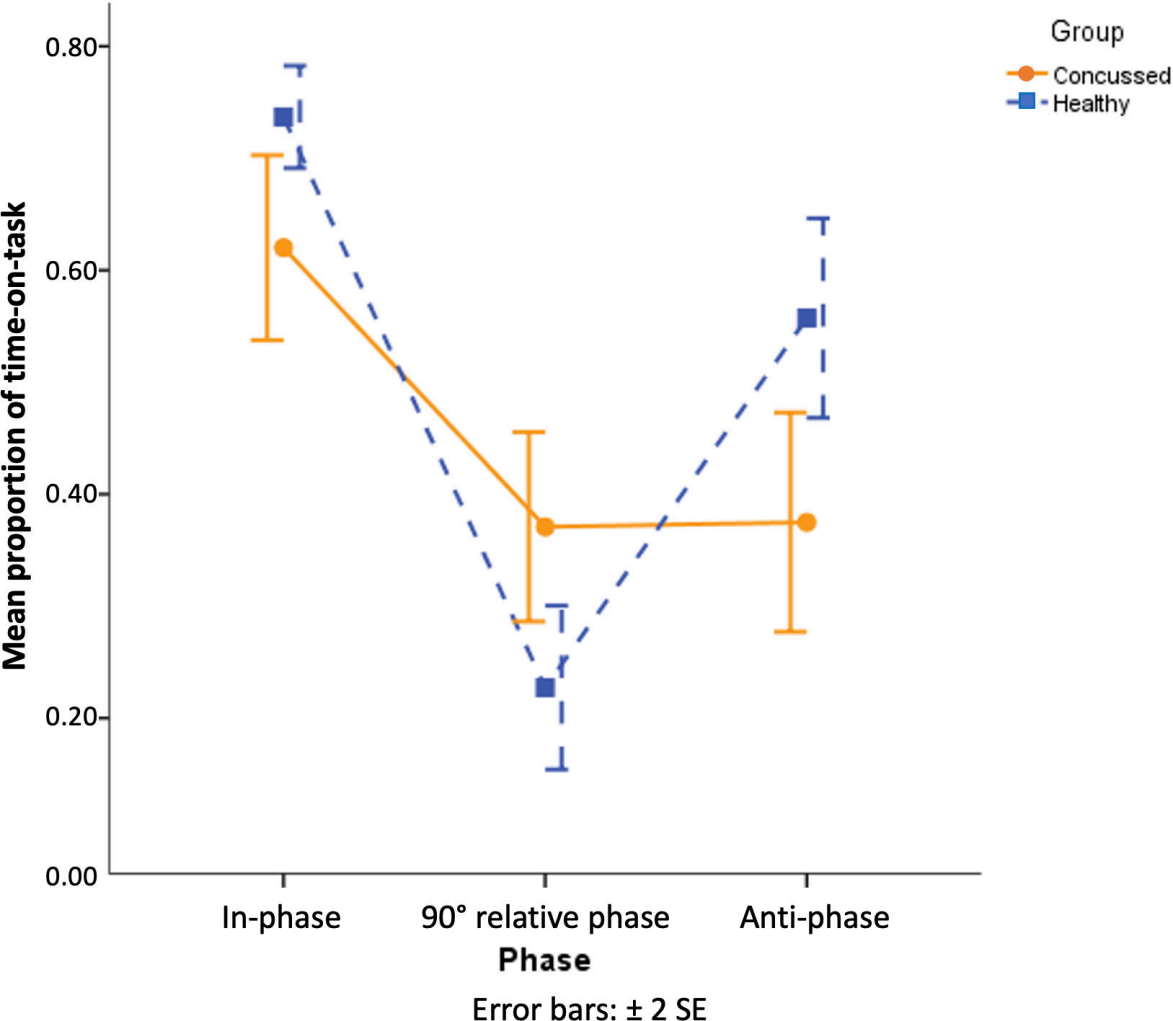
Mean proportion of time-on-task at unimanual in-phase, unimanual 90° relative phase, and unimanual anti-phase for participants with a history of concussion and healthy controls. A conventional pattern of performance (V-shape) was demonstrated by healthy controls but not by the group with a history of concussion. Error bars represent the SE of the mean.

The 2-way mixed-design ANOVA on mean PTTs yielded a main effect for target coordination pattern (*F*_2,36_=61.35, *P*<.001, and Cohen *d*=3.52). As seen in Figure 5, in general, performance of in-phase coordination (mean PTT 0.68, SD 0.12) was significantly better than that of anti-phase coordination (mean PTT 0.47, SD 0.17) and then that of 90° coordination (mean PTT 0.30, SD 0.14). Significant 2-way interaction between group and target coordination pattern was detected (*F*_2,36_=12.60, *P*<.001, and Cohen *d*=1.54). Simple main effect analysis showed that healthy controls outperformed participants with a history of concussion in performing in-phase (*F*_1,54_=4.20, *P*=.045, and Cohen *d*=0.48; mean PTT_Concussed_=0.62, SD 0.13; and mean PTT_Healthy_=0.74, SD 0.07) and anti-phase coordination patterns (*F*_1,54_=10.26, *P*=.002, and Cohen *d*=0.81; mean PTT_Concussed_=0.37, SD 0.15; and mean PTT_Healthy_=0.56, SD 0.14), while the participants with a history of concussion outperformed healthy controls only in performing 90° coordination (*F*_1,54_=6.36, *P*=.015, and Cohen *d*=0.62; mean PTT_Concussed_=0.37, SD 0.13; and mean PTT_Healthy_=0.23, SD 0.12). To be noted, the 2 groups demonstrated different patterns in performing the 3 coordination patterns. As revealed by simple main effect analysis followed by post hoc of Tukey HSD test, healthy controls (*F*_2,54_=41.20, *P*<.001, and Cohen *d*=2.38) performed in-phase coordination (mean PTT 0.74, SD 0.07) significantly better than anti-phase coordination (mean PTT 0.56, SD 0.14), while their performance of 90° coordination (mean PTT 0.23, SD 0.12) was significantly worse than that of both in-phase and anti-phase patterns. However, this differentiated performance between the 3 coordination patterns (as healthy controls showed in Figure 5) was absent for participants with a history of concussion (*F*_2,54_=12.57, *P*<.001, and Cohen *d*=1.27) and their performance of in-phase coordination (mean PTT 0.62, SD 0.13) was better than that of anti-phase (mean PTT 0.37, SD 0.15) and 90° coordination (mean PTT 0.37, SD 0.13), with no difference detected between the latter 2, suggesting that their cognitive-motor system could not differentiate the 2 coordination patterns involving displacement of dots.

## Discussion

### Principal Findings

In experiment 1, three coordination tasks have been implemented to examine the comprehensive cognitive-motor functions of people with and with no history of concussion, and among the 10 task measures, 4 measures have been identified with a great effect size to capture the group difference (Table 2). The results suggest that participants with a history of concussion demonstrated greater variability in perceptual discrimination of the in-phase coordination pattern and worse motor performance in both unimanual and bimanual coordination tasks than healthy controls. These deficits were consistently seen in the following bimonthly sessions, suggesting persistent cognitive-motor impairments in individuals with prior concussion.

Experiment 2 was conducted using an iterated protocol based on the results of experiment 1 and examined the possibility of using the unimanual coordination task alone to detect the cognitive-motor deficits associated with concussion. Compared with the protocol used in experiment 1, the current testing protocol could be completed within 10 minutes (instead of an hour) with the group difference being captured in just 1 testing session. The results showed that unimanual coordination tasks were sensitive to detecting performance deficits in participants with a history of concussion in that individuals with a history of concussion performed worse on both in-phase and anti-phase tasks and failed to differentiate between anti-phase and 90° coordination patterns.

### Visual and Motor Coordination Deficits Revealed Through Comprehensive Assessments

In experiment 1, the visual discrimination task was designed to assess cognitive functions of coordination, which include attending to and tracking the 2 moving dots on the screen, memorizing the spatial-temporal pattern of their movements, and then identifying the target coordination pattern from those distracting patterns. Although these cognitive functions have been reported to be impaired by concussion in previous studies [42-44], participants in this study did not show group differences in discriminating the intrinsic coordination patterns, suggesting that our participants with a history of concussion have recovered the cognitive functions required for perceiving the fundamental coordination tasks such as walking and jumping. This is conforming to the literature that most cognitive dysfunctions associated with concussion spontaneously recover to baseline within 2 weeks [45]. Nevertheless, the analysis on the CV of JNDs revealed that our participants with a history of concussion were more inconsistent in discriminating the in-phase pattern than their healthy counterparts, suggesting that the ability to consistently detect the minor decoupling between 2 oscillators (eg, minor asynchronization of foot takeoff in jumping) was impaired following concussion despite the spontaneous recovery of the ability to perceive intrinsic coordination patterns.

The unimanual coordination task was designed to examine cognitive-motor functions of coordination, specifically, the ability to produce and maintain a rhythmic movement to interact with a computer based on the established perception of coordination pattern. Such an ability not only requires the aforementioned cognitive functions to detect and retain the coordination pattern but also demands motor functions to move one hand in coordination with the moving dot on the screen and correct any perceived movement error to maintain the coordination pattern. The group difference was captured when motor functions were demanded together with cognitive functions. Our healthy controls outperformed participants with a history of concussion in performing both intrinsic coordination patterns. Such a finding conforms to a previous study [46] suggesting that the deficits of dynamic motor functions (eg, visuomotor tracking and walking under various conditions of attention) persist following the concussion despite the spontaneous recovery of cognitive functions. As for the performance of the novel coordination pattern, both participants with a history of concussion and healthy participants improved over the testing sessions without showing any group difference, suggesting that the unimanual coordination task was quite challenging in nature, and the ability to learn new coordination pattern remained intact postconcussion.

Finally, the bimanual coordination task was used to examine the motor functions of coordination. Compared with the unimanual task, the bimanual task demands the motor production of the coordination pattern entirely by requiring participants to move 2 hands rhythmically; therefore, the independent visuomotor control and hemisphere cross talk are both challenged. The results showed the group difference only in performing the in-phase pattern with healthy controls outperforming the participants with a history of concussion. Such a finding not only supported previous studies suggesting that bimanual coordination deficits are associated with concussion [47] but also indicated that the concussion-induced malfunction of CC might have caused the problem for spatial and temporal coupling of 2 hands, which is typically seen in patients with split brain or callosotomy [48]. Compared with the anti-phase and novel coordination patterns, the in-phase pattern demands more spatial and temporal coupling of 2 hands. Therefore, people with a history of concussion are more likely to be screened out by the in-phase bimanual coordination task.

In sum, all 3 coordination tasks have been proven effective for discriminating people with a history of concussion with at least 1 task measure that could be used. Interestingly, most effective task measures are related to the ability to perceive or perform the in-phase pattern, suggesting that the cognitive-motor deficits associated with concussions may be specifically tied to the inability to detect and maintain spatiotemporal coupling in performing eye-hand coordination tasks. The unimanual task emerged to be a good candidate for screening people with a history of concussion because (1) half of effective task measures were found in the unimanual task with the largest effect size to detect group difference, (2) it examines the cognitive-motor functions required for both perceiving and performing coordination patterns, and (3) the performance can be quickly assessed and used for evaluation. Consequently, experiment 2 was designed to examine whether the unimanual task alone can serve as a quick and efficient tool for detecting the chronic cognitive-motor deficits following concussion.

### Perception-Action Coupling Disruptions in Simplified Unimanual Coordination Task

In experiment 2, the unimanual coordination task entails the integration of cognitive-motor functions including attention, memory, and visuomotor tracking, for which one needs to successfully detect the spatiotemporal relationship between the 2 moving dots on the screen and then adjust the manual movement to maintain that pattern. According to the perception-action theory of coordination [29,49], the relative direction of 2 oscillators is used in perceiving and performing both in-phase and anti-phase coordination patterns, while the relative position of 2 oscillators is used for the 90° coordination. Since in-phase and anti-phase patterns represent the coordination movements with the relative direction always being identical (ie, in-phase) or opposite (ie, anti-phase), they are distinctive and easy to be perceived and produced. In contrast, the relative position is ambiguous in 90° coordination due to the fluctuations in the movement direction, which makes it difficult for both perception and production [50]. Both perceptual and motor studies on rhythmic coordination using healthy participants [31,35,38,51] have shown that the performance of 90° coordination is worse than that of in-phase and anti-phase, with the former outperforming the latter (the differentiated performance between 3 coordination patterns as seen in Figure 5 for healthy controls).

In both experiments when unimanual coordination performance was assessed, we found consistently that healthy controls outperformed participants with a history of concussion in producing both in-phase and anti-phase patterns while maintaining the differentiated performance between all 3 coordination patterns, suggesting that they were sensitive to the information of relative direction that is required for perceiving and performing the intrinsic coordination patterns, although having difficulty in finding the information of relative position for successful perception and production of the novel coordination pattern due to lack of practice. As for the participants with a history of concussion, they showed a significantly degraded performance at both in-phase and anti-phase while losing the performance difference between anti-phase and 90°, suggesting that the previous concussion might have limited their ability to discriminate and use the appropriate spatiotemporal information for successful production of both intrinsic and novel coordination patterns. As shown in experiment 1, the participants with a history of concussion did not have a problem with visual discrimination of in-phase and anti-phase patterns; therefore, their degraded performance in performing unimanual in-phase and anti-phase patterns had to do with the deficit in perception-action coupling. Although their performance at 90° was seemingly better than the healthy controls, such a performance was not different from their performance in producing the anti-phase pattern, suggesting that anti-phase and 90° coordination patterns may have been treated as the same by the individuals with a history of concussion since they both involve the displacement and opposite relative direction of moving oscillators. Hence, their seemingly better performance at 90° was a false positive, stemming from their confusion between 90° coordination and anti-phase coordination.

Although more studies are warranted to prove the impaired ability to discriminate and use spatiotemporal information for coordination by the participants with a history of concussion, experiment 2 overall showed that participants with a history of concussion demonstrated deteriorated cognitive-motor functions in performing the unimanual in-phase and anti-phase patterns, which could be attributed to the dissociation between perception and action. In addition, the conventional differentiated performance between the 3 coordination patterns (in-phase > anti-phase > 90°) was not seen for participants with a history of concussion, which suggests that the differentiated performance between the intrinsic and novel coordination patterns in the unimanual coordination task could potentially serve as a useful indicator for screening concussion.

### Limitations and Future Directions

This study has several limitations that should be acknowledged. First, although 2 experiments were conducted, the sample size in each was relatively small, which may limit the generalization of both study findings. While several outcomes demonstrated large effect sizes for group main effect (Cohen *d*>0.80), which suggest a possibility of using a combined approach for assessment and screening, a larger sample size is warranted in future studies to prove the effectiveness and reliability of using eye-hand coordination tasks to detect the chronic or subacute cognitive-motor deficits associated with concussion.

Second, there was a wide range of the reported time from the last concussion. The reported last concussion occurrence ranged from 2 to 96 months in experiment 1 and from 4 to 54 months in experiment 2. As a result, participants could be at different stages of spontaneous recovery from concussion. Also, during that time, participants could be reinjured by concussion that was not medically examined. Future studies could improve on this by focusing on participants within narrower postinjury time frames or by using recovery stage as a covariate in analyses.

Finally, poor eye-hand coordination could result from neurocognitive disorders other than concussion (eg, posttraumatic stress disorder and attention-deficit/hyperactivity disorder). Although all participants in this study were college students with ongoing study programs, and they self-reported to have no history of substance abuse, peripheral neuropathy, and neurological disorders, there is still a possibility for undiagnosed neurocognitive disorders other than concussion to impact the eye-hand coordination performance. In future work, standardized screening tools or neuropsychological assessments could be included to help isolate the impact of concussion from other potential influences on cognitive-motor performance.

### Conclusions

This study provides preliminary evidence for using the computerized eye-hand coordination tasks to detect the chronic cognitive-motor dysfunctions associated with concussion. In developing neurobehavioral tools to capture the postconcussion chronic cognitive-motor deficits, the unimanual coordination task should be considered, and the differentiated performance between intrinsic and novel coordination patterns should be examined.

## Abbreviations

CC: corpus callosum
CV: coefficient of variation
HSD: Honestly Significant Difference
JND: just noticeable difference
mTBI: mild traumatic brain injury
PTT: proportion of time-on-task
